# Anatomical and molecular characterization of dopamine D1 receptor-expressing neurons of the mouse CA1 dorsal hippocampus

**DOI:** 10.1007/s00429-016-1314-x

**Published:** 2016-09-27

**Authors:** Emma Puighermanal, Laura Cutando, Jihane Boubaker-Vitre, Eve Honoré, Sophie Longueville, Denis Hervé, Emmanuel Valjent

**Affiliations:** 1 CNRS UMR 5203, Institut de Génomique Fonctionnelle, 141 rue de la Cardonille, 34094 Montpellier Cedex 05, France; 2 INSERM, U1191, Montpellier, 34094 France; 3 Université de Montpellier, UMR 5203, Montpellier, 34094 France; 4 Inserm, UMR-S 839, 75005 Paris, France; 5 Université Pierre et Marie Curie-Paris 6, 75005 Paris, France; 6 Institut du Fer à Moulin, 75005 Paris, France

**Keywords:** Dopamine D1 receptor, BAC transgenic mice, Interneurons, Hippocampus, RiboTag mice

## Abstract

In the hippocampus, a functional role of dopamine D1 receptors (D1R) in synaptic plasticity and memory processes has been suggested by electrophysiological and pharmacological studies. However, comprehension of their function remains elusive due to the lack of knowledge on the precise localization of D1R expression among the diversity of interneuron populations. Using BAC transgenic mice expressing enhanced green fluorescent protein under the control of D1R promoter, we examined the molecular identity of D1R-containing neurons within the CA1 subfield of the dorsal hippocampus. In agreement with previous findings, our analysis revealed that these neurons are essentially GABAergic interneurons, which express several neurochemical markers, including calcium-binding proteins, neuropeptides, and receptors among others. Finally, by using different tools comprising cell type-specific isolation of mRNAs bound to tagged-ribosomes, we provide solid data indicating that D1R is present in a large proportion of interneurons expressing dopamine D2 receptors. Altogether, our study indicates that D1Rs are expressed by different classes of interneurons in all layers examined and not by pyramidal cells, suggesting that CA1 D1R mostly acts via modulation of GABAergic interneurons.

**Electronic supplementary material:**

The online version of this article (doi:10.1007/s00429-016-1314-x) contains supplementary material, which is available to authorized users.

## Introduction

Beside its crucial role in encoding reward-related events (Schultz 2016), dopamine (DA) also processes salient/non-rewarding signals (Bromberg-Martin et al. 2010). This functional diversity is underlined by the molecular, electrophysiological, and projection-specific heterogeneity of midbrain DA neurons (Lammel et al. 2012; Poulin et al. 2014). For instance, the activation of DA neurons projecting to the lateral shell of the nucleus accumbens triggers reward-associated behaviors while those innervating the medial prefrontal cortex control aversion (Lammel et al. 2012; Poulin et al. 2014). The optimal processing of both rewarding and aversive events also relies on the ability of properly using contextual information (Lisman and Grace 2005). In this context, numerous evidence indicate that midbrain DA neurons projecting to the dorsal hippocampus are activated when animals are exposed to novel environment (Horvitz et al., 1997; Ljungberg et al., 1992), thereby facilitating the encoding of novel contextual cues associated with rewards or potential threats (Bromberg-Martin et al. 2010).

Tract-tracing studies indicate that in the dorsal hippocampus DA neurons originating from the ventral tegmental area (VTA) preferentially innervate CA1 subfields (Broussard et al. 2016; Gasbarri et al. 1997; McNamara et al. 2014; Rosen et al. 2015). Within this area, DA through the stimulation of D1-like receptors has been shown to regulate aversive contextual learning (Broussard et al. 2016; Furini et al. 2014; Heath et al. 2015; Rossato et al. 2009), object-place configuration learning (Furini et al. 2014; Lemon and Manahan-Vaughan 2006) and strength new spatial memories (Bethus et al. 2010; McNamara et al. 2014).

The localization of D1R in the CA1 subfield has been for a long time elusive. *Drd1a*-*EGFP* BAC transgenic mice represent a valuable tool to address this issue (Valjent et al. 2009). The analysis of GFP-positive cells indicates that D1R-expressing neurons populate all CA1 layers and express GAD67, a marker of GABAergic interneurons (Gangarossa et al., 2012). However, the identity of D1R-expressing CA1 GABAergic interneurons among the thirty-seven distinct types identified remains unknown (Wheeler et al. 2015; http://www.hippocampome.org). We therefore conducted a careful examination of the molecular identity of GFP-expressing neurons in the CA1 subfield of *Drd1a*-*EGFP* mice.

## Materials and methods

### Mouse mutants

Male and female, 8–12-week old, *Drd1a*-*EGFP* (*n* = 11 C57BL/6N background, founder *S118*), *Drd2*-*Cre* (C57BL/6J background, founder ER44) heterozygous mice and RiboTag:loxP [The Jackson Laboratory, (Sanz et al., 2009)] were used in this study. BAC *Drd1a*-*EGFP* and *Drd2*-*Cre* mice were generated by GENSAT (Gene Expression Nervous System Atlas) at the Rockefeller University (New York, NY, USA) (Gong et al. 2003). Homozygous RiboTag female mice were crossed with heterozygous *Drd2*-*Cre* male mice to generate *Drd2*-*Cre::RiboTag* mice (Puighermanal et al., 2015). Animals were maintained in a 12 hour light/dark cycle, in stable conditions of temperature and humidity, with food and water ad libitum. All experiments were in accordance with the guidelines of the French Agriculture and Forestry Ministry for handling animals (authorization number/license D34-172-13).

#### Tissue preparation and immunofluorescence

Mice were rapidly anaesthetized with pentobarbital (500 mg/kg, i.p., Sanofi-Aventis, France) and transcardially perfused with 4 % (weight/vol.) paraformaldehyde in 0.1 M sodium phosphate buffer (pH 7.5) (Bertran-Gonzalez et al. 2008). Brains were post-fixed overnight in the same solution and stored at 4 °C. Thirty-μm thick sections were cut with a vibratome (Leica, France) and stored at −20 °C in a solution containing 30 % (vol/vol) ethylene glycol, 30 % (vol/vol) glycerol, and 0.1 M sodium phosphate buffer, until they were processed for immunofluorescence. Hippocampal sections were identified using a mouse brain atlas and sections comprised between −1.34 and −2.06 mm from bregma were included in the analysis (Franklin and Paxinos 2007). Sections were processed as follows: free-floating sections were rinsed three times 10 minutes in Tris-buffered saline (50 mM Tris–HCL, 150 mM NaCl, pH 7.5). After 15 minutes incubation in 0.2 % (vol/vol) Triton X-100 in TBS, sections were rinsed in TBS again and blocked for 1 hour in a solution of 3 % BSA in TBS. Finally, they were incubated 72 hours at 4 °C in 1 % BSA, 0.15 % Triton X-100 with the primary antibodies (Table 1). Sections were rinsed three times for 10 minutes in TBS and incubated for 45–60 minutes with goat Cy2-, Cy3- and Cy5-coupled (1:400, Jackson Immunoresearch) and/or goat alexafluor 488 (1:400, Life Technologies). Sections were rinsed for 10 minutes twice in TBS and twice in Tris-buffer (1 M, pH 7.5) before mounting in 1,4-diazabicyclo-[2. 2. 2]-octane (DABCO, Sigma-Aldrich).

**Table 1:** List of primary antibodies

Antigen	Host	Dilution	Supplier	Catalog no
HA	Mouse	1:1000	Covance	MMS-101R
GFP	Chicken	1:1000	Life Technologies	A10262
CR	Rabbit	1:1000	Swant	7699/3H
CB	Rabbit	1:1000	Swant	CB382
PV	Rabbit	1:1000	Swant	PV25
mGluR1α	Rabbit	1:500	Abnova	PAB14526
NPY	Rabbit	1:500	Abcam	ab10980
SOM	Rabbit	1:300	Millipore	AB5494
nNOS	Mouse	1:300	Sigma	N2280
RLN	Mouse	1:500	Millipore	MAB5364
VGLUT3	Guinea pig	1:500		Gift from El Mestikawy
CB1R	Rabbit	1:1000	Frontier Institute	CB1-Rb-Af380
D2R	Rabbit	1:500	Frontier Institute	D2R-Rb-Af960

*HA* hemagglutinin, *GFP* green fluorescent protein, *PV* parvalbumin, *CB* calbindin-D28k, *CR* calretinin, *NPY* neuropeptide Y, *mGluR1α* metabotropic glutamate receptor type 1α, *SOM* somatostatin, *nNOS* neuronal nitric oxide synthase, *RLN* reelin, *VGLUT3* vesicular glutamate transporter type 3, *CB1R* cannabinoid receptor type 1, *D2R* dopamine D2 receptor

**Fig. 1:**
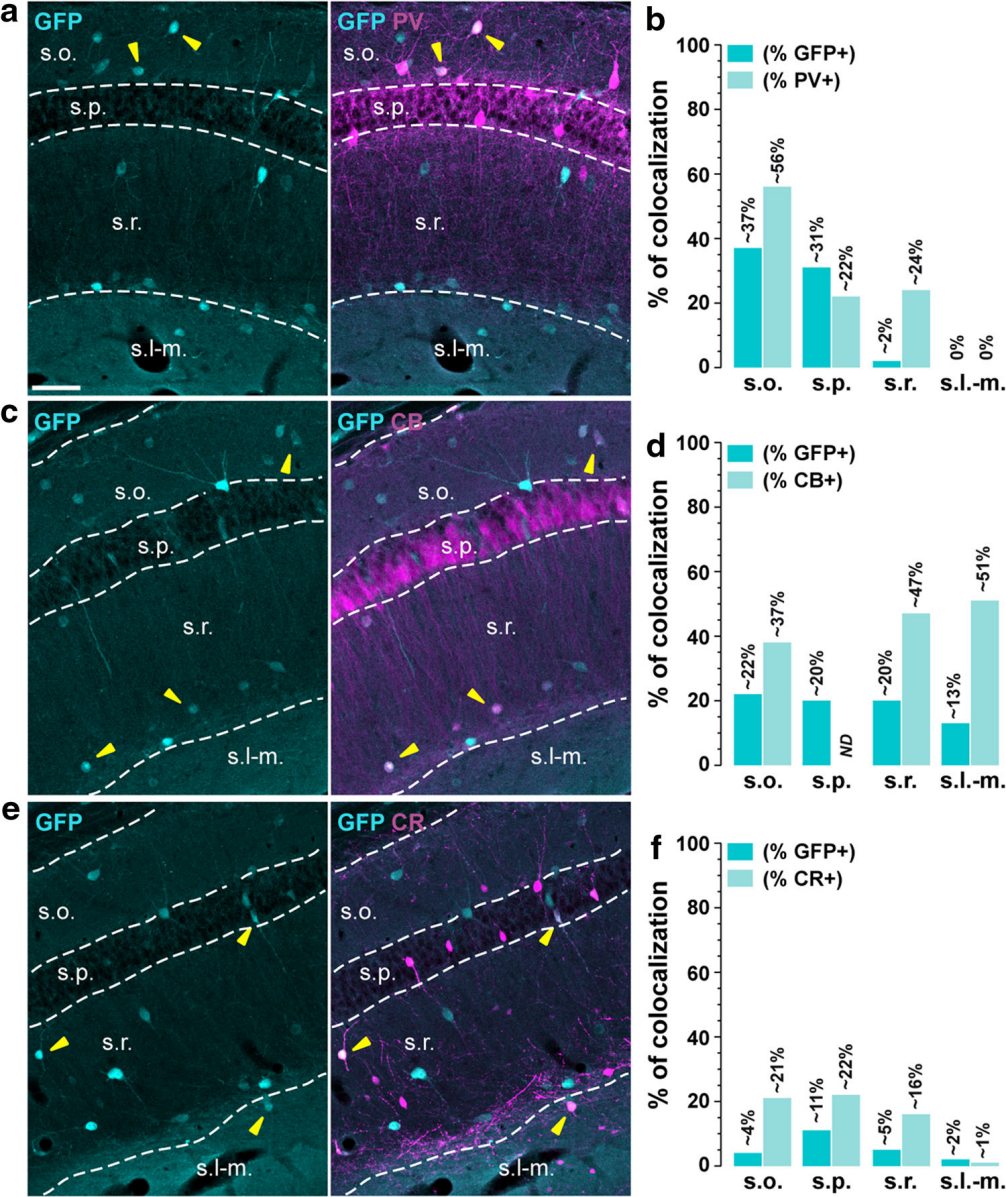
Parvalbumin-, calbindin-D28k-, and calretinin-positive neurons in the dorsal hippocampus in *Drd1a*-*EGFP* mice. **a**, **c**, **e** Single immunofluorescence for GFP (*left panels*) and double immunofluorescence (*right panels*) for GFP (*cyan*) and parvalbumin (*magenta*, PV) (**a**), calbindin-D28k (*magenta*, CB) (**c**), and calretinin (*magenta*, CR) (**e**) in CA1 dorsal hippocampus of *Drd1a*-*EGFP* mice. **a**, **c**, **e** *Yellow arrowheads* indicate GFP/markers-positive neurons. **b**, **d**, **f** Histograms showing the co-expression as percentage of GFP-positive cells (*darkened color*, GFP^+^) and as percentage of cells expressing parvalbumin (*lightened color*, PV^+^) (**b**), calbindin-D28k (*lightened color*, CB^+^) (**d**), and calretinin (*lightened color*, CR^+^) (**f**). Numbers of GFP^+^, PV^+^, CB^+^ and CR^+^ cells counted are reported in Table 2 (4 hemispheres per mouse, 4 mice). *Scale bar* 50 μm

**Fig. 2:**
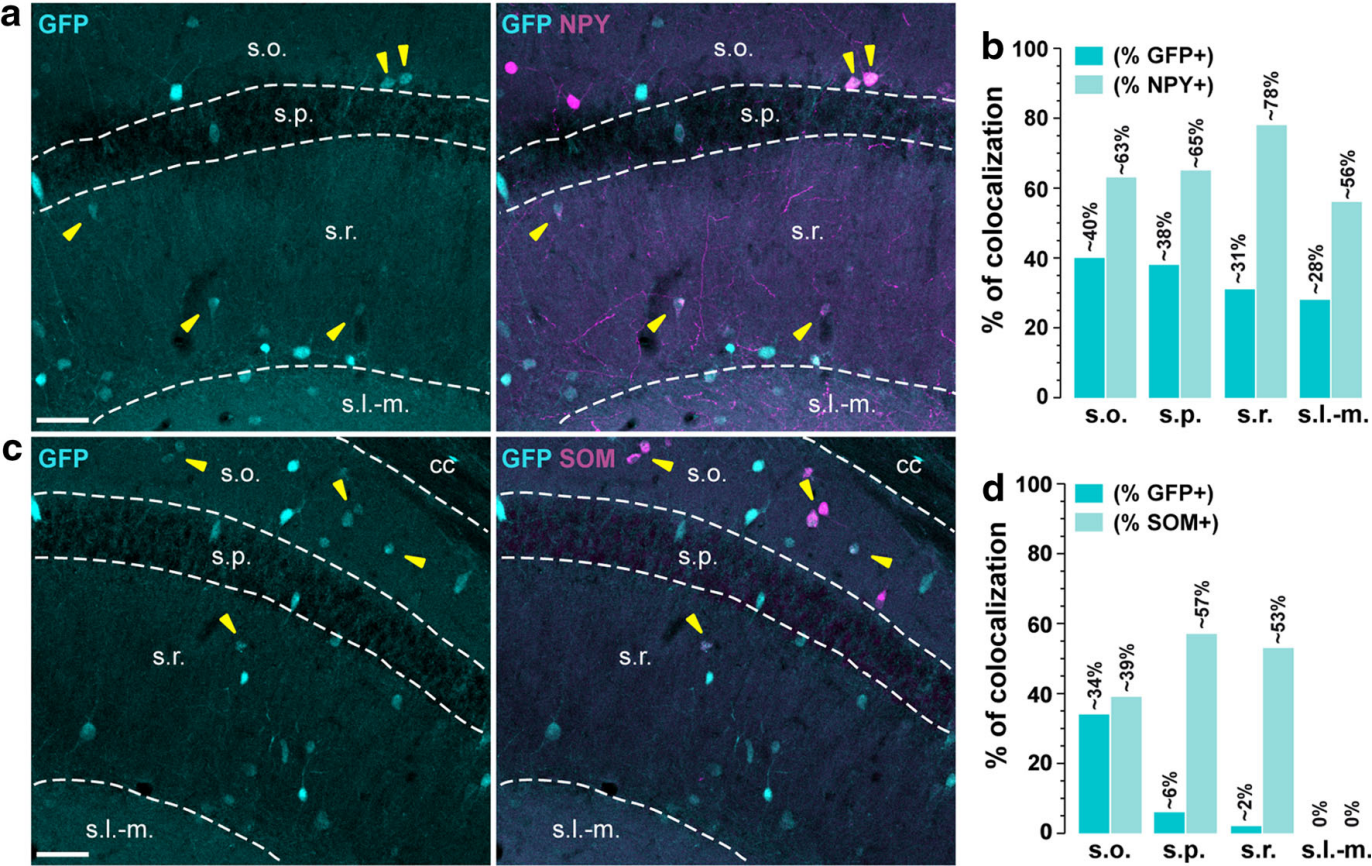
Neuropeptide Y- and somatostatin-expressing cells in the dorsal hippocampus in *Drd1a*-*EGFP* mice. **a**, **c** Single immunofluorescence for GFP (*left panels*) and double immunofluorescence (*right panels*) for GFP (*cyan*) and neuropeptide Y (*magenta*, NPY) (**a**) and somatostatin (*magenta*, SOM) (**c**) in dorsal hippocampus of *Drd1a*-*EGFP* mice. **a**, **c** *Yellow arrowheads* indicate GFP/NPY- or GFP/SOM-positive neurons. **b**, **d** Histograms showing the co-expression as percentage of GFP-positive cells (*darkened color*, GFP^+^) and as percentage of cells expressing NPY (*lightened color*, NPY^+^) (**b**) and somatostatin (*lightened color*, SOM^+^) (**d**). Numbers of GFP^+^, NPY^+^ and SOM^+^ cells counted are reported in Table 2 (4 hemispheres per mouse, 4 mice). *Scale bars* **a**, **c**, 50 μm

**Fig. 3:**
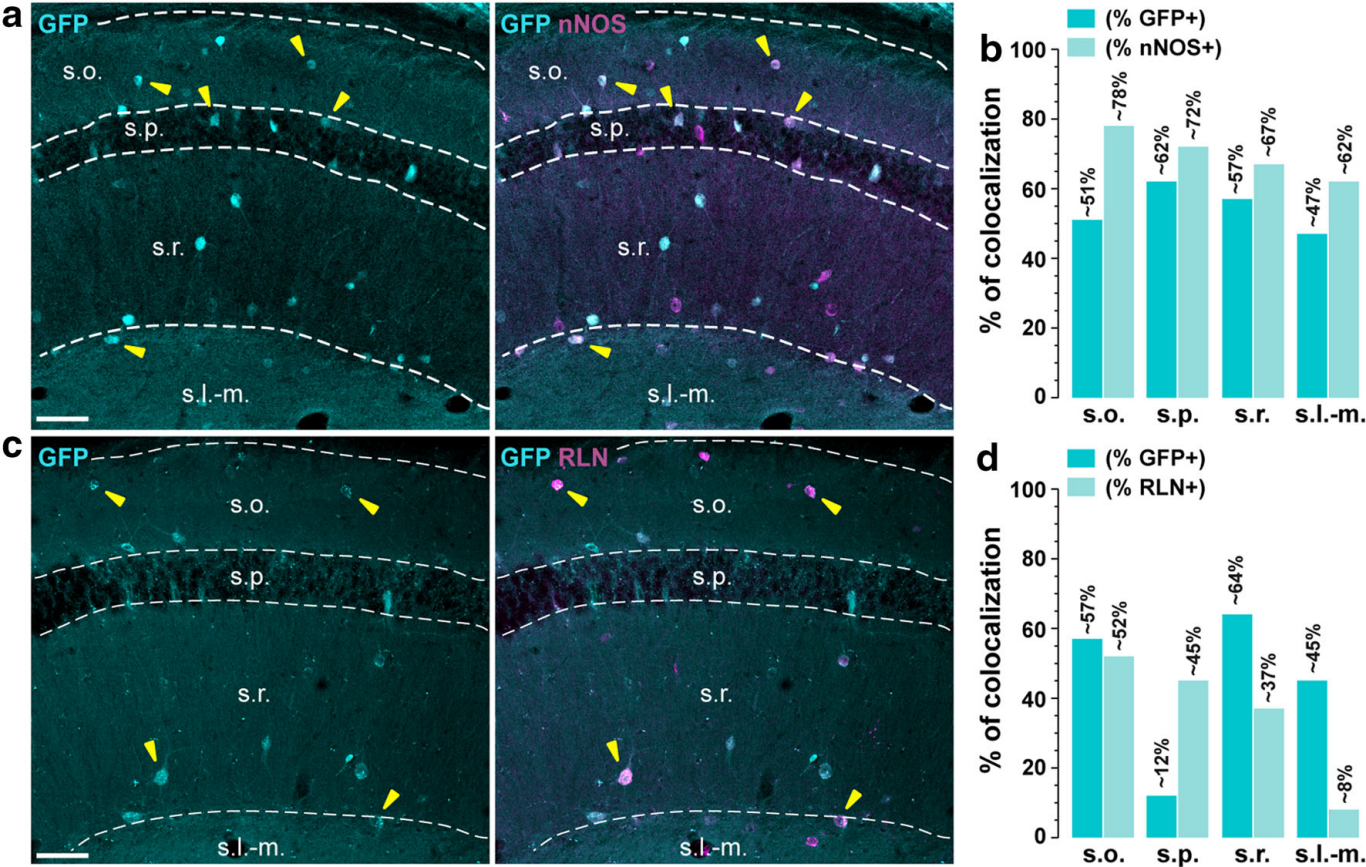
Distribution of D1R-expressing cells among nNOS- and Reelin-positive neurons. **a**, **c** Single immunofluorescence for GFP (*left panels*) and double immunofluorescence (*right panels*) for GFP (*cyan*) and neuronal nitric oxide synthase (*magenta*, nNOS) (**a**) and reelin (*magenta*, RLN) (**b**) in dorsal hippocampus of *Drd1a*-*EGFP* mice. **a**, **c** *Yellow arrowheads* indicate GFP/nNOS- or GFP/RLN-positive neurons. **b**, **d** Histograms showing the co-expression as percentage of GFP-positive cells (*darkened color*, GFP^+^) and as percentage of cells expressing nNOS (*lightened color*, nNOS^+^) (**b**) and reelin (*lightened color*, RLN^+^) (**d**). Numbers of GFP^+^, nNOS^+^ and RLN^+^ cells counted are reported in Table 2 (4 hemispheres per mouse, 3 mice). *Scale bars* **a**, **c**, 50 μm

**Fig. 4:**
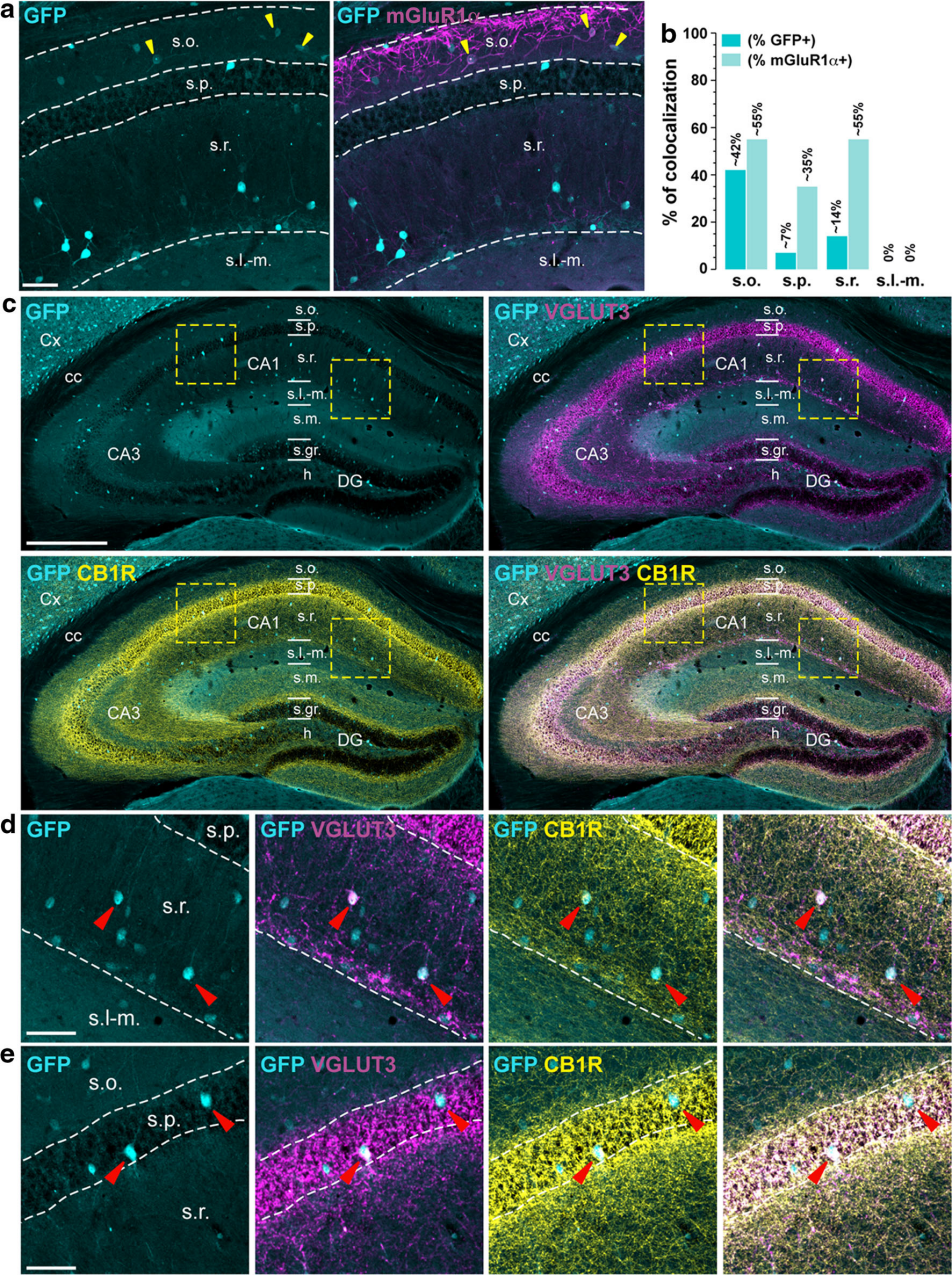
Distribution of D1R-expressing cells among mGluR1α-, CB1R, and VGLUT3-positive neurons. **a** GFP (*cyan*) and mGluR1α (*magenta*) immunofluorescence in the dorsal hippocampus of *Drd1a*-*EGFP* mice. *Yellow arrowheads* indicate GFP/mGluR1α-positive neurons in CA1 subfield *Scale bar* 50 μm. **b** Histograms showing the co-expression as percentage of GFP-positive cells (*darkened color*) and as percentage of cells expressing mGluR1α (*lightened color*). Numbers of GFP^+^ and mGluR1α^+^ cells counted are reported in Table 2 (4 hemispheres per mouse, 4 mice). **c** Triple immunofluorescence for GFP (*cyan*), the vesicular glutamate transporter type 3 (*magenta*, VGLUT3), and the cannabinoid receptor type 1 (*yellow*, CB1R) in the dorsal hippocampus of *Drd1a*-*EGFP* mice. *Scale bar* 400 μm. **d**, **e** High magnification images of areas delineated by the yellow stippled squares. *Red arrowheads* indicate GFP/VGLUT3/CB1R-positive neurons in the *strata radiatum* (**d**) and *pyramidale* (**e**) in CA1 subfield. *Scale bars* **d**, **e**, 60 μm

Confocal microscopy and image analysis were carried out at the Montpellier RIO Imaging Facility. Images covering the entire dorsal hippocampus were single confocal sections acquired using sequential laser scanning confocal microscopy (Zeiss LSM780). Double-labeled images from each region of interest were single section obtained using sequential laser scanning confocal microscopy (Zeiss LSM780). Photomicrographs were obtained with the following band-pass and long-pass filter setting: alexafluor 488/Cy2 (band pass filter: 505–530), Cy3 (band pass filter: 560–615) and Cy5 (long-pass filter 650). Figure 1, 2, 3, 4, and 5: GFP labeled neurons were pseudocolored cyan and markers immunoreactive neurons were pseudocolored magenta. From the overlap of cyan and magenta, double-labeled neurons appeared white. Figure 4: GFP- and VGLUT3-labeled neurons were pseudocolored cyan and magenta and CB1R-positives fibers were pseudocolored yellow. Images used for quantification were all single confocal sections. GFP- and markers-positive cells were manually counted in the CA1 area taking into account the laminar location. Cells were considered positive for a given marker only when the nucleus was clearly visible. Adjacent serial sections were never counted for the same marker to avoid any potential double counting of hemisected neurons. Values in the histograms in Figures represent the co-expression as percentage of GFP-positive cells (darkened color) and as percentage of cells expressing the various markers tested in each laminar location in the CA1 subfield (6–12 hemispheres, *n* = 3–4 mice). Total numbers of GFP- and marker-positive cells counted are reported in Table 2.

**Fig. 5:**
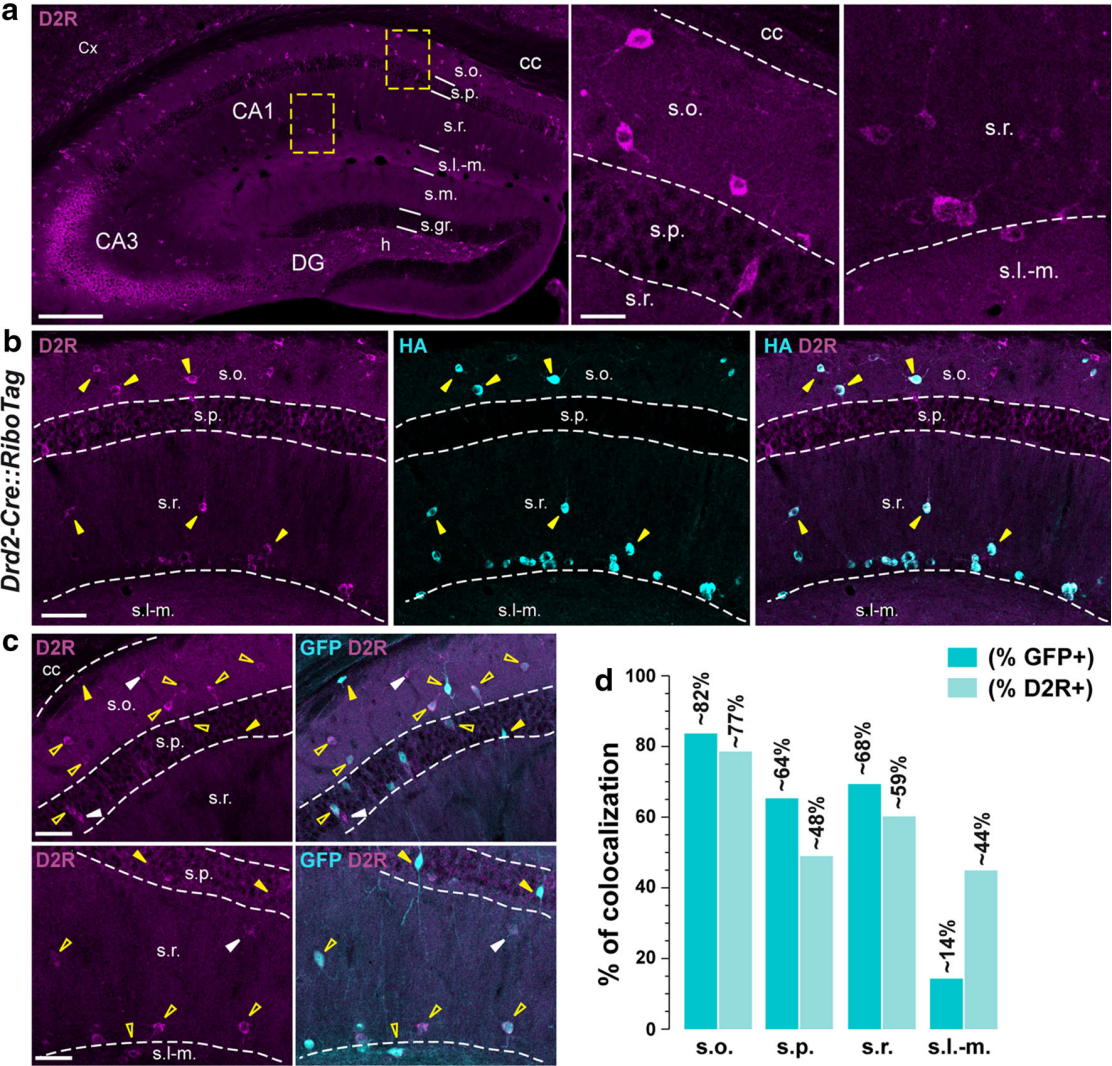
Dopamine D2R-positive neurons in the CA1 dorsal hippocampus of *Drd1a*-*EGFP* mice. **a** D2R immunofluorescence in the dorsal hippocampus of *Drd1a*-*EGFP* mice. High magnification images of areas delineated by the yellow stippled squares in CA1 subfield. *Scale bars * 400 and 20 μm. **b** HA (*cyan*) and D2R (*magenta*) immunofluorescence in the dorsal hippocampus in *Drd2*-*Cre::RiboTag* mice. *Yellow arrowheads* indicate HA/D2R-positive neurons in the CA1 subfield. Note that all HA-expressing cells are also D2R-positive. *Scale bar* 60 μm. **c** GFP (*cyan*) and D2R (*magenta*) immunofluorescence in the dorsal hippocampus of *Drd1a*-*EGFP* mice. *Yellow open arrowheads* indicate GFP/D2R-positive neurons, *yellow arrowheads* indicate GFP-expressing cells, and *white arrowheads* indicate D2R-positive neurons. *Scale bar* 60 μm. **d** Histograms showing the co-expression as percentage of GFP-positive cells (*darkened color*) and as percentage of cells expressing D2R (*lightened color*). Numbers of GFP^+^ and D2R^+^ cells counted are reported in Table 2 (4 hemispheres per mouse, 3 mice)

**Table 2:** Number of cells quantified in the dorsal CA1 mouse hippocampus

Figures	GFP/markers	s.o.	s.p.	s.r.	s.l-m
Figure 1b	GFP	411	288	421	145
	PV	271	400	37	5
	GFP/PV	153	89	9	0
Figure 1d	GFP	372	272	421	139
	CB	217	ND	177	35
	GFP/CB	82	55	84	18
Figure 1f	GFP	428	286	476	173
	CR	76	144	154	261
	GFP/CR	16	31	25	3
Figure 2b	GFP	469	294	564	208
	NPY	297	171	223	104
	GFP/NPY	188	111	174	58
Figure 2d	GFP	401	216	396	138
	SOM	351	21	17	0
	GFP/SOM	136	12	9	0
Figure 3b	GFP	214	141	244	94
	nNOS	141	123	206	71
	GFP/nNOS	110	88	139	44
Figure 3d	GFP	212	123	137	65
	RLN	229	33	235	359
	GFP/RLN	120	15	87	29
Figure 4b	GFP	353	229	371	68
	mGluR1α	271	43	95	0
	GFP/mGluR1α	149	15	52	0
Figure 5d	GFP	220	108	216	110
	D2R	236	143	247	34
	GFP/D2R	181	69	146	15

*GFP* green fluorescent protein, *PV* parvalbumin, *CB* calbindin-D28k, *CR* calretinin, *NPY* neuropeptide Y, *SOM* somatostatin, *nNOS* neuronal nitric oxide synthase, *RLN* reelin, *mGluR1α* metabotropic glutamate receptor type 1α, *D2R* dopamine D2 receptor, *ND* not determined

#### Polyribosome immunoprecipitation

HA-tagged-ribosome immunoprecipitation was performed as described previously (Sanz et al. 2009) with slight modifications. The hippocampus from *Drd2*-*Cre::RiboTag* mice was homogenized by douncing in 1-ml polysome buffer (50 mM Tris, pH 7.4, 100 mM KCl, 12 mM MgCl2, and 1 % NP-40 supplemented with 1 mM DTT, 1 mg/ml heparin, 100 μg/ml cycloheximide, 200 U/ml RNAseOUT, and protease inhibitor mixture). Samples were then centrifuged at 10,000×*g* for 10 minutes to collect the postmitochondrial supernatant. Then, 100 μl of each supernatant was transferred to a new tube serving as input fraction for validation. Anti-HA antibody (5 μl/sample; Covance, #MMS-101R) was added to the remaining supernatant and incubated overnight at 4 °C with constant gently rotation. The following day, samples were added to protein G magnetic beads (Invitrogen, #100.04D) and incubated overnight at 4 °C with constant gently rotation. On the third day, magnetic beads were washed twice in a magnetic rack for 10 minutes each in high-salt buffer (50 mM Tris, pH 7.4, 300 mM KCl, 12 mM MgCl2, 1 %NP-40, 1 mM DTT, and 100 μg/ml cycloheximide). After washing, 350 μl of Qiagen RLT buffer (supplemented with β-Mercaptoethanol) were added to the pellets and to the input samples. RNA was extracted according to manufacturer’s instructions using a Qiagen RNeasy Micro kit and quantified using Nanodrop 1000 spectrophotometer.

#### cDNA synthesis and quantitative real-time PCR

Synthesis of cDNA was performed on input fraction (10 % of homogenate) and pellet fraction (after HA immunoprecipitation), which were reverse transcribed to first strand cDNA using the SuperScript^®^ VILO™ cDNA synthesis kit (Invitrogen). Resulting cDNA was used for quantitative real-time PCR (qRT-PCR), using SYBR Green PCR master mix on the LC480 Real-Time PCR System (Roche) and the primer sequences listed in Table 3. Analysis was performed using LightCycler^®^ 480 Software (Roche). Data are expressed as the fold change comparing the pellet fraction versus the input (3 biological replicates per set of primers). The immunoprecipitated RNA samples (pellet) were compared to the input sample in each case.

**Table 3:** Sequences of PCR primers

Marker	PCR primers
*Gfap*	*Sense*, AGCGAGCGTGCAGAGATGA
	*Antisense*, AGGAAGCGGACCTTCTCGAT
*Cnp1*	*Sense*, GCTGCACTGTACAACCAAATTCTG
	*Antisense*, ACCTCCTGCTGGGCGTATT
*Iba1*	*Sense*, CCCCCAGCCAAGAAAGCTAT
	*Antisense*, GCCCCACCGTGTGACATC
*Camk2a*	*Sense*, TTTGAGGAACTGGGAAAGGG
	*Antisense*, CATGGAGTCGGACGATATTGG
*Slc1a1*	*Sense*, AAAGATAGCAGGAAGGTAACCGAAT
	*Antisense*, CGGTCAGTCGGTAGCTTTCAG
*Calb2*	*Sense*, TGAGAATGAACTGGACGCCCTC
	*Antisense*, GTAGAGCTTCCCTGCCTCGG
*Gad1*	*Sense*, TTGTGCTTTGCTGTGTTTTAGAGA
	*Antisense*, CCCCCTGCCCAAAGATAGAC
*Sst*	*Sense*, CTGTCCTGCCGTCTCCAGTG
	*Antisense*, CTCTGTCTGGTTGGGCTCGG
*Slc32a1*	*Sense*, TCACGACAAACCCAAGATCAC
	*Antisense*, GTCTTCGTTCTCCTCGTACAG
*Drd2*	*Sense*, CTCTTTGGACTCAACAACACAGA
	*Antisense*, AAGGGCACGTAGAACGAGAC
*Drd1a*	*Sense*, TCGAACTGTATGGTGCCCTT
	*Antisense*, TGGGGTTCAGGGAGGAATTC

#### Statistical analysis

Unpaired Student’s *t-*test was used to compare changes in gene expression between inputs and pellets. Significance threshold was set at *p* < 0.05. Prism 6.0 software was used to perform statistical analyses.

## Results

### Distribution of D1R-expressing cells among calcium-binding proteins

*Parvalbumin* (*PV*). PV-positive cells are widely distributed in the CA1 subfield (Fig. 1a). Depending on their location in the different layers they allow the classification of various GABAergic inhibitory interneurons (Klausberger 2009; Pawelzik et al. 2002). Thus, PV-expressing cells identified axo-axonic, basket, and bistratified interneurons in both *strata pyramidale* and *oriens*. In this latter layer, it also marked the horizontal axo-axonic and oriens-lacunosum-moleculare (O-LM). Our analysis revealed that GFP/PV-positive cells represented ~37 and ~31 % of the total GFP labeled in *strata oriens* and *pyramidale*, respectively (Fig. 1a, b; Table 2). Low or no co-localization was found in *strata radiatum* (~2 %) and *lacunosum*-*moleculare* (0 %) where PV-positive cells identified perforant path-associated QuadD, quadrilaminar, and R-receiving apical targeting interneurons (Fig. 1a, b; Table 2).

*Calbindin*-*D28k* (*CB*). In CA1 subfield, CB-immunoreactivity is found in both principal glutamatergic cells in *strata pyramidale* and *radiatum* as well as in GABAergic interneurons located in *strata oriens*, *radiatum,* and *lacunosum*-*moleculare* (Jinno and Kosaka 2002) (Fig. 1c). In *stratum pyramidale*, we found that among the 272 GFP-immunoreactive cells quantified, 55 co-localized with CB (~20 % of total of GFP-positive neurons) (Fig. 1c, d; Table 2). In *stratum oriens*, where CB-positive cells identified recurrent O-LM, oriens alveus, and SO–SO cells, ~22 % of GFP-labeled neurons co-expressed CB (Fig. 1c, d). Finally, CB immunolabeling also marked LMR-projecting, radiatum, and Schaffer collateral associated classes of interneurons in *strata radiatum* and *lacunosum*-*moleculare*, in which ~20 and ~13 % of CB/GFP-positive neurons were detected (Fig. 1c, d; Table 2).

*Calretinin* (*CR*). CA1 CR-positive cells are distributed in all the layers where they allow the identification of several classes of interneurons (Wheeler et al. 2015) (Fig. 1e). Overall, our analysis revealed a low degree of co-localization between GFP and CR immunoreactivity whatever the layers analyzed. The highest percentage of co-localization was found in *stratum pyramidale* (~11 %) where CR-positive cells marked interneuron specific LMO-O, interneuron specific O-targeting QuadD, interneuron specific R-O, and interneuron RO-O, a class of interneurons specialized in the control of other interneurons (Fig. 1e, f; Table 2). In addition to interneurons specific, CR-positive cells were expressed in oriens-bistratified in *stratum oriens* and perforant path-associated QuadD, quadrilaminar, and Schaffer collateral receiving R-targeting cells in *stratum radiatum*. In both layers, GFP-positive cells expressing CR was rather low, representing only ~4 % in *stratum oriens* and ~5 % in *stratum radiatum* (Fig. 1e, f; Table 2). Finally, in *stratum* *lacunosum*-*moleculare* where CR cells stain Cajal–Retzius cells and quadrilaminar interneurons, only three GFP/CR-positive cells were detected among the 173 GFP-immunoreactive cells (Fig. 1e, f; Table 2). These co-labeled cells which most likely correspond to quadrilaminar interneurons represented only ~2 % (Fig. 1e, f; Table 2).

### Distribution of D1R-expressing cells among neuropeptides

*Neuropeptide Y* (*NPY*). NPY/GFP-positive neurons were found in all the layers of the CA1 subfield (Tricoire et al. 2011). Co-localized GFP and NPY immunoreactive cells represented ~40 % and of ~38 % of GFP-positive cells in *strata oriens* and *pyramidale*, respectively. Within these two layers, NPY marked back-projection, O-LM, recurrent O-LM, SO–SO interneurons as well as bistratified and ivy cells (Fig. 2a, b; Table 2). Co-localization was also high in *strata radiatum* (~31 %) and *lacunosum*-*moleculare* (~28 %) in which ivy, LMR, perforant path-associated QuaD, radiatum, and radial trilaminar interneurons as well as neurogliaform interneurons are distributed (Fig. 2a, b; Table 2).

*Somatostatin* (*SOM*). The highest percentage of GFP/SOM-positive neurons was detected in *stratum oriens* (~34 %) where SOM is expressed by several classes of interneurons including O-LM, recurrent O-LM, O-LMR, oriens-bistratified, oriens-bistratified projecting as well as trilaminar (Chittajallu et al. 2013; Tricoire et al. 2011) (Fig. 2c, d; Table 2). In *stratum pyramidale*, only ~6 % of GFP-positive cells co-expressed SOM, a marker of bistratified interneurons. It should be noted that a significant fraction (~57 %) of these neurons appeared to express D1R. Finally, in *strata radiatum* where SOM-containing cells identify LMR, perforant path-associated QuaD, quadrilaminar and radiatum interneurons, GFP/SOM co-expressing cells represented only ~2 % of GFP-positive cells, but 53 % of SOM-positive neurons (Fig. 2c, d; Table 2). No co-labeling was found in *stratum lacunosum*-*moleculare* (Fig. 2d; Table 2).

### Distribution of D1R-expressing cells among miscellaneous markers

*Neuronal nitric oxide synthase* (*nNOS*). nNOS-expressing neurons represent one of the largest subclasses of interneurons present in the CA1 subfield of the hippocampus. Highly concentrated in *strata oriens* and *lacunosum*-*moleculare*, they allow the identification of neurogliaform and ivy interneurons (Armstrong et al. 2012; Price et al. 2005; Tricoire et al. 2010). As shown in Fig. 3, percentages of nNOS/GFP-immunoreactive cells were high in all the CA1 layers reaching ~62 and ~57 % in *strata pyramidale* and *radiatum*, and being slightly lower in *strata oriens* and *lacunosum*-*moleculare* (~51 and ~47 %, respectively) (Fig. 3a, b; Table 2).

*Reelin* (*RLN*). In CA1 subfield, RLN allows the identification of both glutamatergic and GABAergic interneurons (Wheeler et al. 2015). Our analysis revealed that GFP was never found in small RLN-positive cells located at the border of *strata radiatum/lacunosum*-*moleculare*, which correspond to glutamatergic Cajal–Retzius cells. In contrast, a high level of co-localization was found in *strata oriens* (~57 %), *radiatum* (~64 %), and *lacunosum*-*moleculare* (~45 %) where RLN identified O-LM and neurogliaform interneurons (Fig. 3c, d; Table 2). In *stratum pyramidale*, RLN-immunoreactive cells represented only ~12 % of GFP-expressing neurons (Fig. 3d; Table 2).

### Distribution of D1R-expressing cells among receptors/transporters

*Metabotropic glutamate receptor type 1α (mGluR1α)*. The largest density of mGluR1α-positive cells was found in *stratum oriens* (Tricoire et al. 2011). Within this layer, mGluR1α marked preferentially trilaminar, recurrent O-LM, and O-LM interneurons and co-expressed within GFP in ~42 % of the case (Fig. 4a, b; Table 2). mGluR1α/GFP-expressing cells were also found to a lesser extent in *stratum radiatum* (~14 %), where they identify hippocampo-subicular projecting ENK^+^ interneurons (Fig. 4a, b; Table 2). Low (~7 %) and no co-localization were detected in *strata pyramidale* and *lacunosum*-*moleculare,* respectively (Fig. 4a, b; Table 2).

*Cannabinoid type 1 receptor * (*CB1R*). CA1 CB1R-expressing interneurons are preferentially found in *strata radiatum* and *lacunosum*-*moleculare* identifying LMR projecting, Schaffer collateral-associated, and trilaminar interneurons. They also correspond to CCK-positive basket cells distributed in *strata oriens, pyramidale,* and *radiatum* (Freund and Buzsaki 1996) (Fig. 4c). Because CB1R are mainly presynaptically expressed, hippocampal CB1R immunoreactivity did not allow us to quantify the percentage of CB1R-positive cells among the D1R-expressing population. However, a few scattered CB1R/GFP-positive cells were clearly identified in *stratum radiatum* and at the border of *strata radiatum/lacunosum*-*moleculare* (Fig. 4d) as well as in *stratum pyramidale* (Fig. 4e).

*Vesicular glutamate transporter type 3* (*VGLUT3*). Only four different types of interneurons located in *strata oriens, pyramidale,* and *radiatum* express VGLUT3 (Wheeler et al. 2015). Among them, two classes of VGLUT3-expressing interneurons are also CB1R-positive. These include CCK-positive basket and radial trilaminar interneurons. The two other subtypes are negative for CB1R and identify perforant path-associated QuaD and horizontal basket interneurons. As shown in Fig. 4, a dense plexus of VGLUT3-immunoreactive fibers surrounding the *stratum pyramidale* was detected in the CA1 subfield. Interestingly, most of the sparse VGLUT3/GFP-positive cells detected in *strata radiatum* and *pyramidale* were also positive for CB1R (Fig. 4c–e).

### CA1 D1R-positive cells express dopamine D2 receptors

The present analysis of the distribution of GFP in *Drd1a*-*EGFP* mice suggests that diverse classes of GABAergic interneurons express D1R. Because the distribution of D1R-expressing cells was reminiscent to the one recently described for CA1 D2R-containing neurons (Puighermanal et al. 2015), we analyzed whether GFP/D2R co-expressing cells were present in the CA1 dorsal hippocampus of *Drd1a*-*EGFP*. The analysis of endogenous D2R distribution, using anti-D2R antibody (see Table 1), revealed a pattern of expression of D2R-positive cells that resembles to the one recently described (Puighermanal et al. 2015). Indeed, in the dentate gyrus most of the D2R-positive neurons were located in the hilus identifying the hilar mossy cells (Fig. 5a). In the CA1 subfield, D2R-labeled cells were predominantly detected in *strata oriens* and *radiatum* (Fig. 5a). In addition, an intense D2R immunoreactivity was detected in *stratum lacunosum*-*moleculare* most likely corresponding to the terminals of O-LMs interneurons (Fig. 5a). On the other hand, they were rarely found in *stratum pyramidale* (Fig. 5a). The pattern of distribution of endogenous D2R-expressing cells was further confirmed by analyzing the degree of co-localization between D2R and HA immunoreactivity in *Drd2*-*Cre::RiboTag* mice. As illustrated, all HA-expressing cells located in *strata oriens, radiatum,* and at the border of *strata radiatum/lacunosum*-*moleculare* were also positive for D2R (Fig. 5b, yellow arrows). Only a few neurons were D2R^+^/HA^−^ suggesting that the expression of endogenous D2R might not be fully recapitulated in *Drd2*-*Cre::RiboTag* mice (Fig. 5b).

We next examined the degree of co-localization of GFP-labeled cells with D2R in the dorsal CA1 hippocampus of *Drd1a*-*EGFP*. In *stratum oriens* a large majority of GFP-expressing cells were also D2R positive (~82 %). The percentage of GFP/D2R-immunoreactive neurons was also high in *strata pyramidale* and *radiatum*, reaching ~64 and ~68 %, respectively (Fig. 5c, d; Table 2). By contrast, in *stratum lacunosum*-*moleculare* only 15 GFP/D2R-positive cells have been detected among the 110 GFP-immunoreactive cells (Fig. 5c, d; Table 2).

**Fig. 6:**
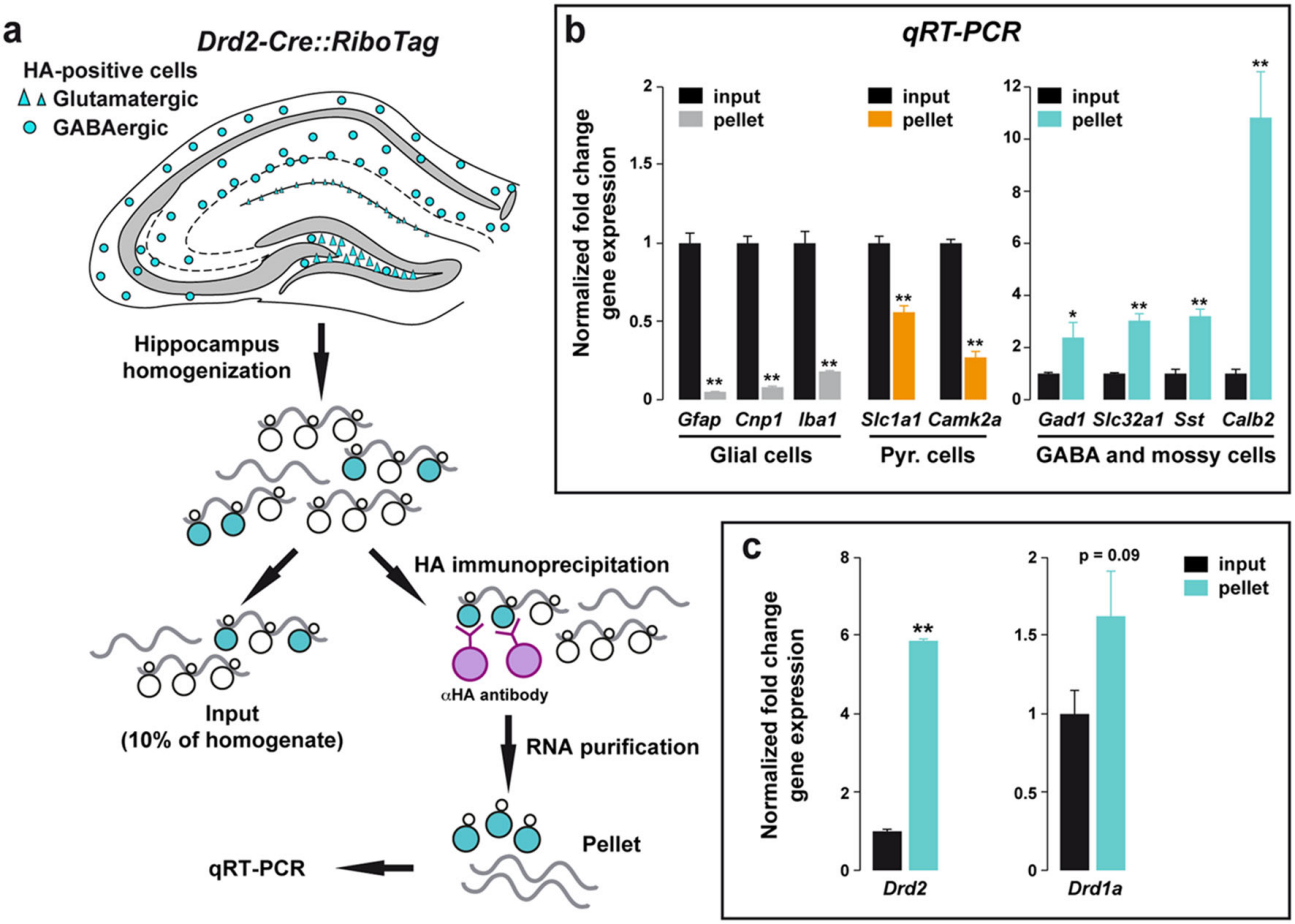
D2R-expressing cells are enriched in *Drd1* mRNA. **a** D2R-expressing cells, either glutamatergic (*triangles*) or GABAergic (*circles*), contain ribosomes tagged with the HA epitope in *Drd2*-*Cre::RiboTag* mice. After hippocampus homogenization, 10 % of the lysate was saved as input fraction (containing all mRNAs), while the mRNAs bound to tagged-ribosomes were isolated through HA-immunoprecipitation (pellet fraction). **b** Quantitative RT-PCR analysis of mRNAs isolated following HA immunoprecipitation from hippocampi of *Drd2*-*Cre::RiboTag* mice. All genes were normalized to β-actin. Data are expressed as the fold change comparing the pellet fraction versus the input. Negative control genes, including glial markers (*Gfap*, *Cnp* and *Iba1; grey bars*) and pyramidal cell markers (*Slc1a1* and *Camk2a; orange bars*) were de-enriched in the pellet samples, whereas the positive control genes including GABAergic and mossy cell markers (*Gad1*, *Slc32a1*, *Sst* and *Calb2; cyan bars*) were enriched in the pellet compared to the input fraction. **c** Quantitative RT-PCR analysis of *Drd2* and *Drd1* genes after HA immunoprecipitation from hippocampi of *Drd2*-*Cre::RiboTag* mice (*n* = 6 mice). Data are analyzed by two tailed Student *t* test. *p < 0.05, **p < 0.001 pellet vs. input

To further confirm that both receptors were expressed at least by a fraction of hippocampal cells, we took advantage of the *Drd2*-*Cre::RiboTag* mice that express tagged-ribosomes selectively in D2R-containing cells (Fig. 6a). After homogenization of the hippocampus, tagged-ribosomes and their bound mRNAs were captured by HA immunoprecipitation (Fig. 6a). The analysis by quantitative RT-PCR (qRT-PCR) of purified mRNAs compared to the input fraction revealed a de-enrichment of glial markers, including *Gfap* for astrocytes, *Cnp1* for oligodendrocytes, and *Iba1* for microglia as well as of glutamatergic pyramidal cells markers such as *Camk2a* and *Slc1a1* (EAAT3) (Fig. 6b). By contrast, the glutamatergic Cajal–Retzius and hilar mossy cells marker *Calb2* (CR) was highly enriched in mRNAs purified following HA immunoprecipitation (Fig. 6b). Similarly, GABAergic markers including *Gad1*, *Slc32a1* (VIAAT), and *Sst* (SOM) were also clearly enriched (Fig. 6b) confirming our previous observations (Puighermanal et al. 2015). Finally, the presence of *Drd2* mRNA was confirmed as expected, but also *Drd1a* mRNAs were isolated following HA immunoprecipitation (Fig. 6c), in agreement with the co-localization of *GFP* and *D2R* in *Drd1a*-*EGFP* mice (Fig. 5). Taken together, these results indicate that in CA1 subfield a large proportion of D1R-expressing cells also contain D2R.

## Discussion

Although the mesohippocampal DA pathway has been characterized almost three decades ago (Gasbarri et al. 1994a, b; Swanson 1982), the mechanisms by which DA mediates its effect in the hippocampus remain largely unknown. The precise characterization of hippocampal cells expressing DA receptors is therefore a critical step to understand the functional consequences of DA transmission within the hippocampus. By using BAC transgenic mice expressing EGFP under the control of the D1R promoter, the present study examined the laminar distribution and determined the molecular identity of CA1 D1R-containing cells in the dorsal hippocampus. As initially reported, GFP-labeled neurons were found in all CA1 layers and were essentially GABAergic interneurons (Gangarossa et al. 2012). Our analysis revealed that GFP-positive cells were co-immunolabeled with several neurochemical markers, suggesting that various classes of GABAergic interneurons expressed D1R (Wheeler et al. 2015). Finally, we provide evidence that a large proportion of D1R-expressing neurons located in all CA1 layers also express D2R.

### Anatomical distribution of DA projections and expression pattern of D1R-expressing cells

An early tract-tracing study and double immunofluorescence analyses reported that VTA DA neurons projecting to the hippocampus were preferentially localized in *strata oriens* and *pyramidale*, with sparse fibers in *stratum radiatum* and barely any innervation of *stratum lacunosum*-*moleculare* (Gasbarri et al. 1994a, b; Kwon et al. 2008). This heterogeneous laminar distribution of DA fibers within CA1 was recently confirmed by analyzing hippocampal efferents from genetically defined VTA DA neurons using Cre-inducible AAV-expressing ChR2-EYFP (Broussard et al. 2016; McNamara et al. 2014; Rosen et al. 2015). Interestingly, our analysis revealed that D1R-expressing cells are found in a large proportion in *stratum oriens* and to a lesser extent in *stratum pyramidale*. Thus, in these two layers DA terminals largely overlap with D1R-containing neurons as illustrated by the presence of GFP-labeled neurons in the vicinity of TH-positive fibers (Supplemental Figure 1, Inset). D1R-expressing cells are also present in *strata radiatum* and at the border of *radiatum/lacunosum*-*moleculare*. However, despite the detection of TH immunoreactivity (Supplemental Figure 1), VTA DA neurons do not innervate these two layers (Broussard et al. 2016; McNamara et al. 2014; Rosen et al. 2015). In fact, strong evidence indicate that the dense plexus of TH-labeled fibers observed within *strata radiatum* and *lacunosum*-*moleculare* corresponds to noradrenergic (NE) axons arising from the locus coeruleus (LC) (Kwon et al. 2008). Of interest, a recent study showed that these LC NE fibers co-release DA and NE, suggesting that they constitute the only source of DA in the vicinity of D1R-expressing neurons located in these two layers (Smith and Greene 2012; Walling et al. 2012). Therefore, depending on their laminar location, D1R-expressing neurons could be controlled by DA arising from two distinct sources: the VTA for *strata oriens* and *pyramidale* and the LC for *strata radiatum* and *lacunosum*-*moleculare*.

### Reliability of the distribution of D1R-expressing cells in *Drd1a*-*EGFP* mice

If the expression of D1R by the granule cells in the dentate gyrus is well admitted and has been demonstrated by in situ hybridization, binding, and immunofluorescence studies (Boyson et al. 1986; Fremeau et al. 1991; Gangarossa et al. 2012; Huang et al. 1992; Mansour et al. 1990, 1991; Rocchetti et al. 2015; Sarinana et al. 2014), the distribution/identity of D1R-expressing cells in the CA1 subfield remains unclear. Indeed, although the presence of D1R in pyramidal cells has been suggested (Huang et al. 1992; Kern et al. 2015; Ladepeche et al. 2013a, b), little or no signal for D1R at the transcript level was detected (Fremeau et al. 1991; Mansour et al. 1990; Rocchetti et al. 2015; Sarinana et al. 2014). Consistent with these latter findings, our analysis revealed a weak and sparse distribution of GFP-labeled neurons in *stratum pyramidale*. This finding strongly suggests that the D1R staining detected in this layer and initially thought to label the plasma membrane of CA1 pyramidal cells (Huang et al. 1992) most likely corresponds to D1R-positive terminals arising from another cell type, possibly GABAergic interneurons. Supporting this hypothesis, D1R-expressing cells were found in *strata oriens* and *radiatum/lacunosum*-*moleculare* (Gangarossa et al. 2012; present study), three layers populated by a large diversity of GABAergic interneurons (Wheeler et al. 2015). The distribution of GFP-labeled cells reported in the present study is consistent with the early description of the D1R expression pattern. Thus, although at low density, autoradiography studies revealed the presence of D1R binding sites in *stratum oriens* (Mansour et al. 1990, 1991). Moreover, cells containing D1R mRNA have been detected in both *strata oriens* and *radiatum* (Fremeau et al. 1991). Finally, both layers also exhibit a strong D1R immunoreactivity (Huang et al. 1992). Combined with previous findings, our results strongly suggest that D1R are preferentially expressed by GABAergic interneurons and not by pyramidal cells within the CA1 subfield.

### D1R is expressed in various classes of GABAergic interneurons in CA1

The use of different neurochemical markers including calcium-binding proteins (parvalbumin, calbindin-D28k, calretinin), neuropeptides (somatostatin, NPY), receptors/transporters (mGluR1α, CB1R, VGLUT3) and miscellaneous markers (nNOS, reelin) allowed us to evaluate the distribution of GFP among 33 out of the 37 types of interneurons known to be present in CA1 (Wheeler et al. 2015). Based on the laminar localization and the percentage of co-localization, we estimate that D1R are expressed by at least eight distinct classes of GABAergic interneurons. For instance, the high percentage of GFP/nNOS-positive cells in all layers indicate that D1R might be expressed by both ivy and neurogliaform cells (Armstrong et al. 2012; Price et al. 2005; Tricoire et al. 2010). Their presence in this latter population is further supported by the strong percentage of GFP/NPY- and GFP/reelin-positive neurons estimated in the *stratum lacunosum*-*moleculare* (Fuentealba et al. 2008; Tricoire et al. 2011). In *stratum oriens*, the co-expression of GFP with SOM/mGluR1α suggests that D1R are expressed by O-LMs and trilaminar interneurons (Chittajallu et al. 2013; Klausberger 2009; Matyas et al. 2004; Tricoire et al. 2011). The presence of GFP/PV-expressing cells also favors the hypothesis that axo-axonic, basket, and bistratified interneurons contain D1R. This observation is further strengthened by the recent demonstration of the critical role played by D1R signaling in PV cells for the consolidation of long-term memory (Karunakaran et al. 2016). Finally, although not quantified, the presence of GFP/VGLUT3/CB1R-positive neurons suggests that basket CCK^+^ might express D1R (Wheeler et al. 2015). Interestingly, most of the D1R-containing cells located in the *stratum pyramidale* correspond to GABAergic interneurons. Based on the combination of the molecular marker they express, one can reasonably conclude they comprise axo-axonic, basket, bistratified, and ivy cells. Finally, a small fraction of GFP-positive neurons were co-labeled with calbindin-D28k, confirming the scarity of pyramidal neurons expressing D1R. Our cross analysis also allowed us to identify CA1 cell types devoid of D1R. Thus, at least two types of interneurons, the perforant path-associated QuaD located in *stratum radiatum* and the quadrilaminar interneurons found in both *strata radiatum* and *lacunosum*-*moleculare* (Pawelzik et al. 2002; Tricoire et al. 2011). Finally, the lack of co-localization between GFP and calretinin/reelin-positive cells localized in *stratum lacunosum*-*moleculare* supports the absence of D1R in Cajal–Retzius cells (Marchionni et al. 2010; Tricoire et al. 2011). Further experiments using double fluorescent in situ hybridization and/or cell-type specific mRNA profiling should help to further confirm the presence of D1R transcripts in these distinct classes of GABAergic interneurons.

### Evidence for D1R and D2R co-expression in CA1 GABAergic interneurons

Although BAC transgenic mice expressing fluorescent proteins represent a useful tool to characterize genetically identified cell populations, caution should be taken when analyzing the expression pattern. Indeed, during the course of the characterization of our *Drd2*-*Cre::RiboTag* mouse line, which express tagged-ribosomes selectively in D2R-containing cells, we found that in the hippocampus D2R-expressing cells displayed a much widespread pattern than the one initially described in *Drd2*-*EGFP* mice (Gangarossa et al. 2012; Puighermanal et al. 2015). The difference was particularly evident in CA1 where HA-positive cells of *Drd2*-*Cre::RiboTag* mice identified diverse classes of GABAergic interneurons (Gangarossa et al. 2012; Puighermanal et al. 2015). This observation, together with our present analysis, led us to re-examine whether, in the CA1 subfield, D1R and D2R-expressing cells were fully segregated, as initially thought (Gangarossa et al. 2012; Puighermanal et al. 2015), or could partially overlap. The present findings argue in favor of this last hypothesis. Thus, in strata *oriens, pyramidale*, and *radiatum*, our double immunofluorescence analysis revealed a high degree of co-localization between GFP and D2R. The presence of cells co-expressing both D1R and D2R was further confirmed by the enrichment of *Drd1* transcripts isolated from tagged-ribosomes expressed in D2R-containing cells. Interestingly, the presence of both receptors on diverse GABAergic interneurons, which for some of them have antagonistic activity onto CA1 pyramidal cells, could account for the complexity and variability of DA action following bath application in hippocampal slices. Indeed, while DA bath application does affect excitatory Schaffer collateral (SC) drive onto CA1 pyramidal cells, a depression of the synaptic transmission of temporoammonic (TA) pathway has been reported (Ito and Schuman 2007; Otmakhova and Lisman 1999). This latter effect, which requires both D1R and D2R, also involves local GABAergic interneurons located at the border of *strata radiatum* and *lacunosum*-*moleculare* (Ito and Schuman 2007; Otmakhova and Lisman 1999). However, at this synapse, following high-frequency stimulation, DA facilitates excitatory drive to CA1 pyramidal cells mainly through the decreased feedforward inhibition (Ito and Schuman 2007; Otmakhova and Lisman 1999). Therefore, one can envision that the ability of DA to gate TA synaptic transmission would not only depend on the excitatory inputs frequency but also depend on the dual action of DA on GABAergic interneurons co-expressing both D1R and D2R. Because these different types of D1R/D2R-expressing interneurons innervate specific and distinct domains of pyramidal cells and other interneurons, future experiments will be necessary to understand whether their pattern of activity will change depending on tonic, phasic, or ramping DA signals.

In conclusion, our study revealed that in the CA1 subfield of the hippocampus, distinct classes of GABAergic interneurons express D1R. Contrasting with the dorsal striatum where D1R and D2R are highly segregated (Bertran-Gonzalez et al. 2010; Valjent et al. 2009), a high degree of D1R-containing neurons also express D2R. Future studies using cell-type specific invalidation of D1R and/or D2R are promptly required to untangle the complexity of DA signals within the hippocampus.

## Electronic supplementary material

Below is the link to the electronic supplementary material.

Supplemental Figure 1: TH-positive fibers in the CA1 subfield in *Drd1a*-*EGFP* mice. GFP (cyan) and tyrosine hydroxylase (magenta, TH) immunofluorescence in the hippocampal CA1 of *Drd1a*-*EGFP* mice. Scale bar, 60 μm. Inserts are high magnification images of areas delineated by the yellow stippled rectangle and show TH-positive fibers that made appositions with nearby GFP-expressing cells. Scale bar, 10 μm. (TIFF 3393 kb)

## Abbreviations

Cx: Cortex
DG: Dentate gyrus
cc: *Corpus callosum*
s.o.: *Stratum oriens*
s.p.: *Stratum pyramidale*
s.r.: *Stratum radiatum*
s.l.: *Stratum lucidum*
s.l.-m.: *Stratum lacunosum*-*moleculare*
s.m.: *Stratum moleculare*
o-s.m.: Outer two thirds of the *stratum moleculare*
i-s.m.: Inner-third of the *stratum moleculare*
s.gr.: *Stratum granulosum*
h: Hilus
EGFP: Enhanced green fluorescent protein
HA: Hemagglutinin
CB: Calbindin-D28k
CR: Calretinin
PV: Parvalbumin
NPY: Neuropeptide Y
SOM: Somatostatin
nNOS: Neuronal nitric oxide synthase
RLN: Reelin
VGLUT3: Vesicular glutamate transporter type 3
D1R: Dopamine D1 receptor
D2R: Dopamine D2 receptor
CB1R: Cannabinoid type 1 receptor
mGluR1α: Metabotropic glutamate receptor type 1α
