# Supplementary figures and images for: Anatomical and molecular characterization of dopamine D1 receptor-expressing neurons of the mouse CA1 dorsal hippocampus
Source: Brain Struct Funct. 2016 Sep 27;222(4):1897–911. doi: 10.1007/s00429-016-1314-x (PMC5406422; doi:10.1007/s00429-016-1314-x)

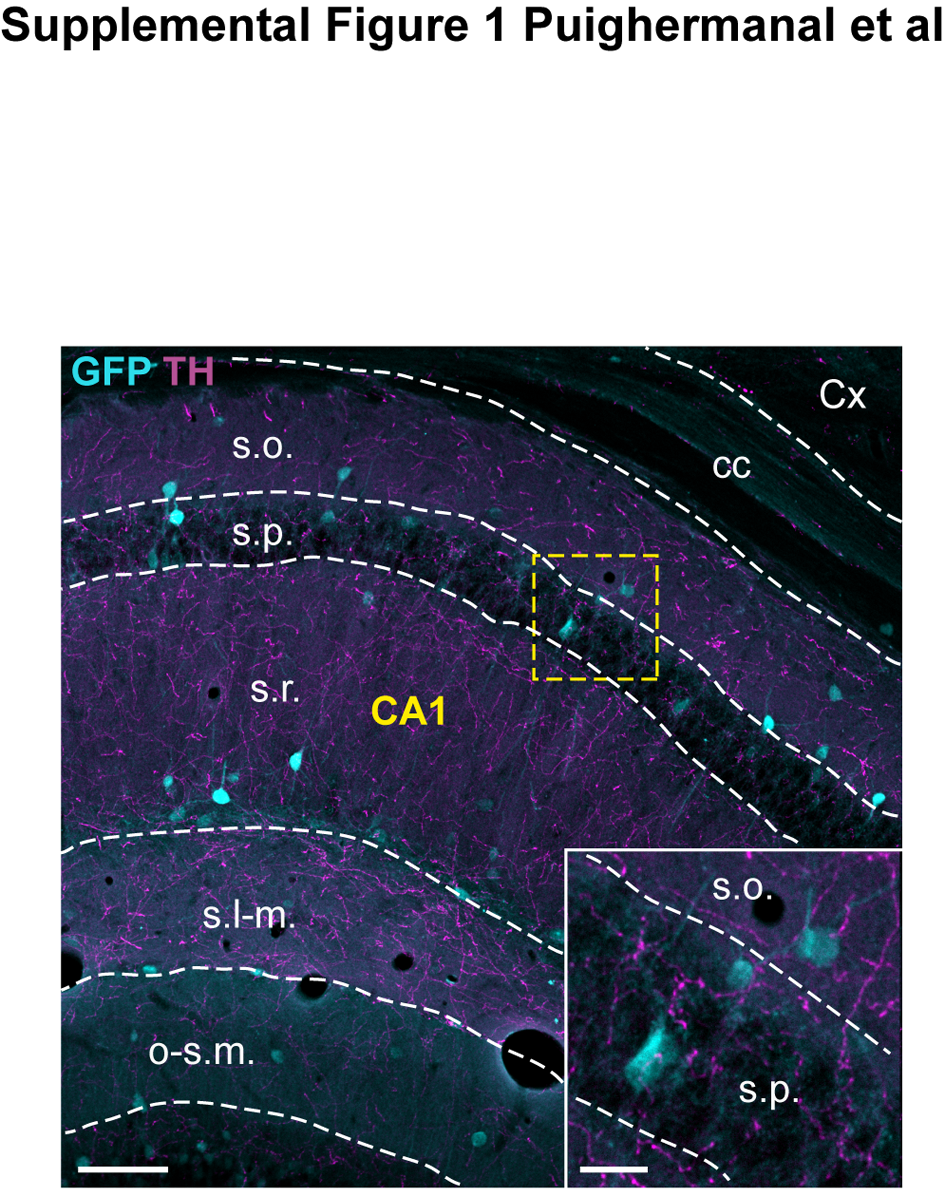

Supplement: Supplementary file 1 — Supplemental Figure 1: TH-positive fibers in the CA1 subfield in Drd1a-EGFP mice. GFP (cyan) and tyrosine hydroxylase (magenta, TH) immunofluorescence in the hippocampal CA1 of Drd1a-EGFP mice. Scale bar, 60 μm. Inserts are high magnification images of areas delineated by the yellow stippled rectangle and show TH-positive fibers that made appositions with nearby GFP-expressing cells. Scale bar, 10 μm. (TIFF 3393 kb) [file 429_2016_1314_MOESM1_ESM.tif]
